# Supplementary figures and images for: Human formin FHOD3-mediated actin elongation is required for sarcomere integrity in cardiomyocytes
Source: eLife. 2025 Jul 15;13:RP104048. doi: 10.7554/eLife.104048 (PMC12263155; doi:10.7554/eLife.104048)

Flipped left to right

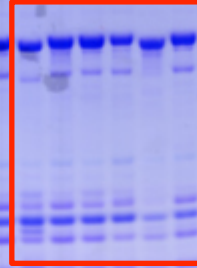

Supplement: Figure 1—figure supplement 1—source data 1. [file elife-104048-fig1-figsupp1-data1.pdf]

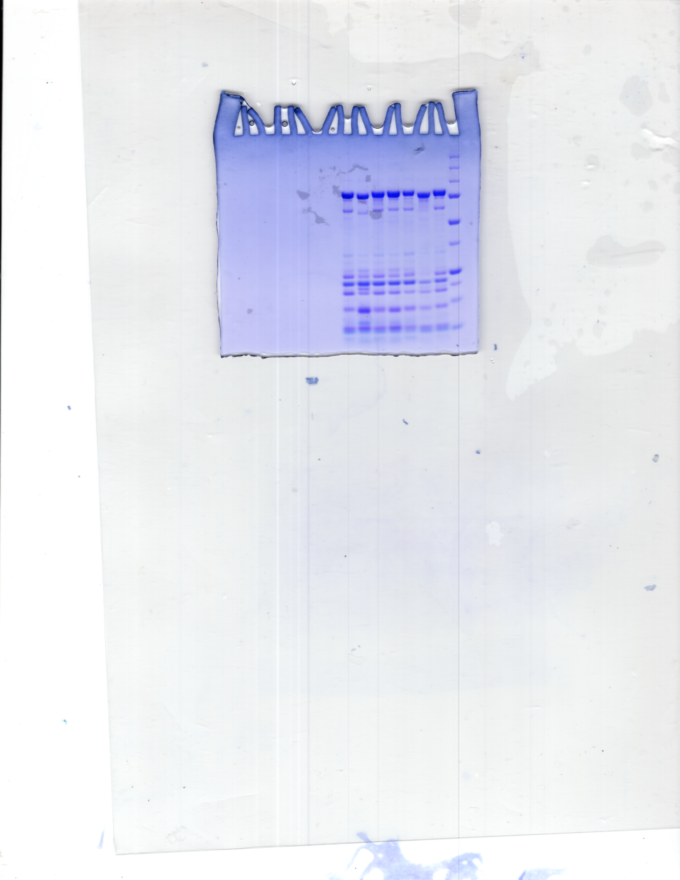

Supplement: Figure 1—figure supplement 1—source data 2. [file elife-104048-fig1-figsupp1-data2.tif]

Figure 2

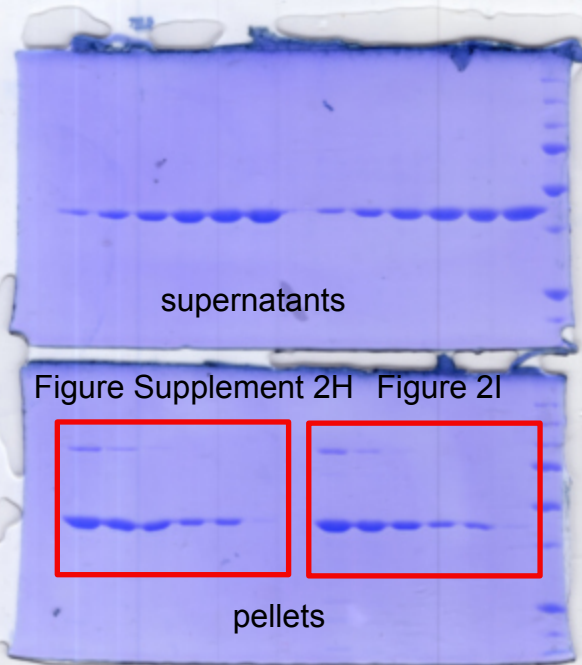

Supplement: Figure 2—source data 1. [file elife-104048-fig2-data1.pdf]

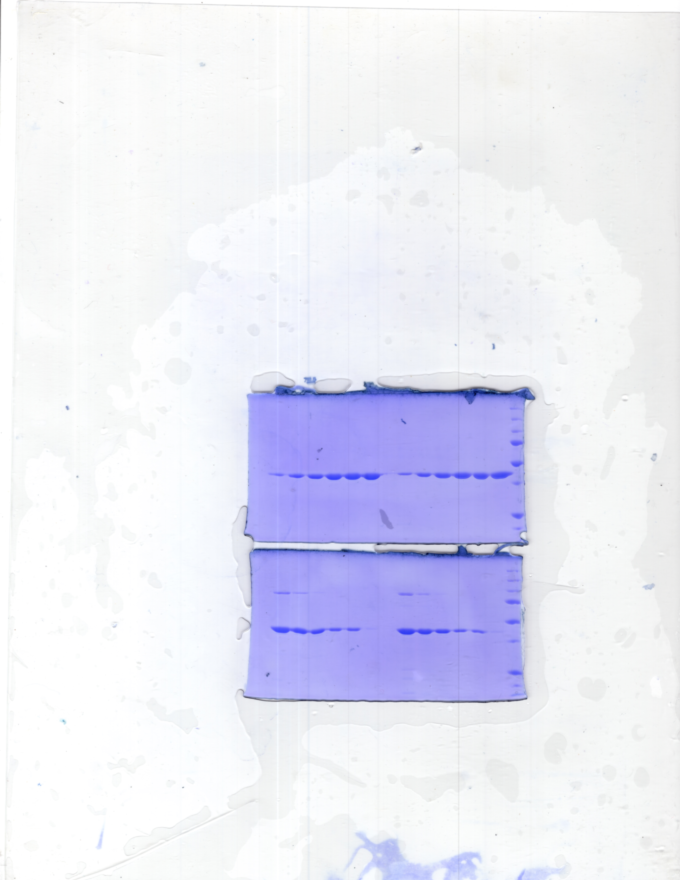

Supplement: Figure 2—source data 2. [file elife-104048-fig2-data2.tif]

Figure 4B

HA

FHOD3

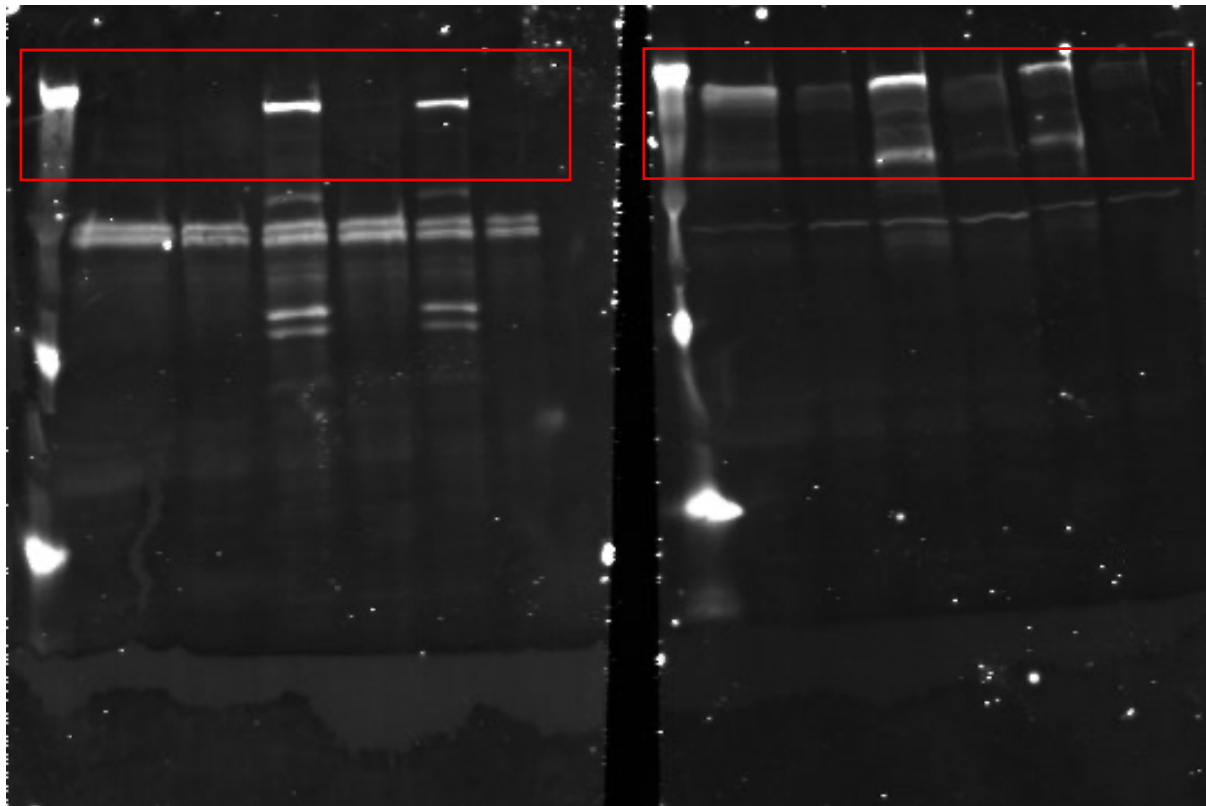

Supplement: Figure 4—source data 1. [file elife-104048-fig4-data1.zip › Figure4B - Source Data 1 labeled.pdf]

Figure 4B: GAPDH

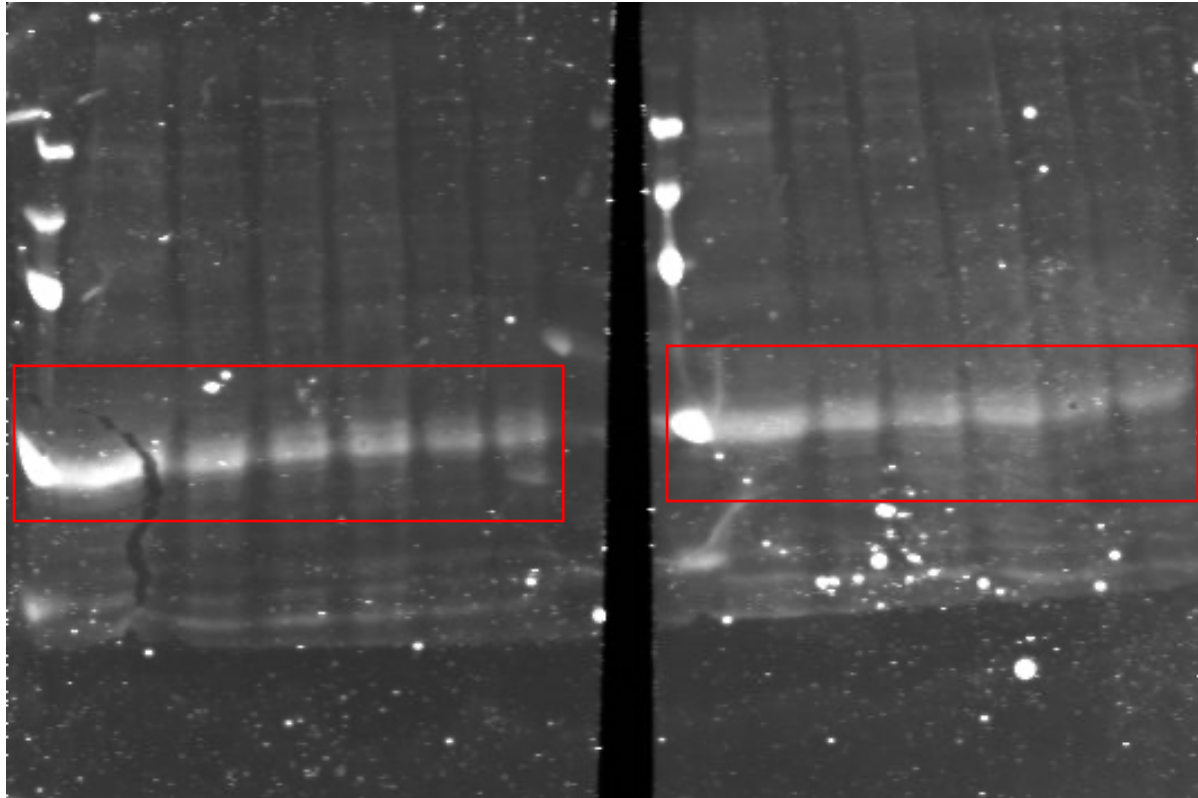

Supplement: Figure 4—source data 1. [file elife-104048-fig4-data1.zip › Figure4B - Source Data 2 labeled.pdf]

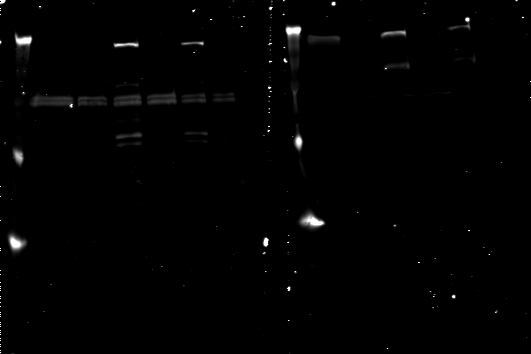

Supplement: Figure 4—source data 2. [file elife-104048-fig4-data2.zip › Figure 4 - Source Data 1.TIF]

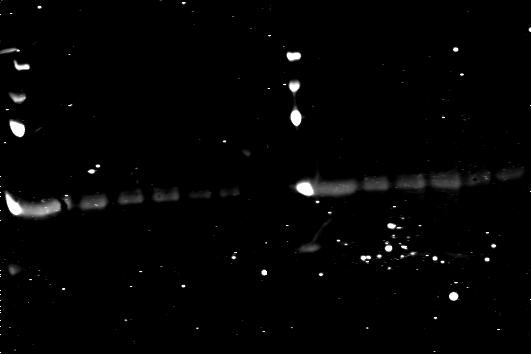

Supplement: Figure 4—source data 2. [file elife-104048-fig4-data2.zip › Figure 4 - Source Data 2.TIF]
